# Spontaneous Occurrence of Gangrene Due to *Clostridium septicum* in a Patient With Advanced Endometrial Carcinoma

**DOI:** 10.1155/S1064744994000372

**Published:** 1994

**Authors:** David H. Moore, Kris Ghosh, Gregory P. Sutton

**Affiliations:** Department of Obstetrics and Gynecology Indiana University Medical Center 550 North University Boulevard UH #2440 Indianapolis IN 46202-5274 USA

## Abstract

*Background:* We report the first known case of spontaneous, atraumatic *Clostridium septicum* gangrene occurring in a patient with recurrent endometrial adenocarcinoma.

*Case:* A 63-year-old white female undergoing chemotherapy for recurrent endometrial adenocarcinoma presented with right “arthritis-like” shoulder pain. She denied fever, chills, or shoulder trauma. The patient was afebrile and her blood pressure was 100/50. Her right shoulder and upper extremity were remarkable for an area of dark blue discoloration with crepitus. The white blood cell (WBC) count was 8,200/μl with left shift. Serum creatinine, platelet count, and coagulation studies were normal. Computed tomography revealed gas in the right shoulder tissues. A Gram stain of fluid aspirated from the shoulder demonstrated gram-positive spore-forming rods. She declined surgical intervention and expired within hours of admission. Cultures of the right shoulder eventually grew *Clostridium septicum.*

*Conclusion:* It is imperative to consider clostridial gangrene in the differential diagnosis for any patient with cancer and a fever of unknown origin.


Infectious Diseases in Obstetrics and Gynecology 2:34–37 (1994)
© 1994 Wiley-Liss, Inc.

### Spontaneous Occurrence of Gangrene Due to *Clostridium septicum* in a Patient With Advanced Endometrial Carcinoma

**David H. Moore, Kris Ghosh, and Gregory P. Sutton**

*Department of Obstetrics and Gynecology, Indiana University Medical Center, Indianapolis, IN*

ABSTRACT

***Background:*** **We report the first known case of spontaneous, atraumatic *Clostridium septicum* gangrene occurring in a patient with recurrent endometrial adenocarcinoma.**

***Case:*** **A 63-year-old white female undergoing chemotherapy for recurrent endometrial adenocarcinoma presented with right "arthritis-like" shoulder pain. She denied fever, chills, or shoulder trauma. The patient was afebrile and her blood pressure was 100/50. Her right shoulder and upper extremity were remarkable for an area of dark blue discoloration with crepitus. The white blood cell (WBC) count was 8,200/µl with left shift. Serum creatinine, platelet count, and coagulation studies were normal. Computed tomography revealed gas in the right shoulder tissues. A Gram stain of fluid aspirated from the shoulder demonstrated gram-positive spore-forming rods. She declined surgical intervention and expired within hours of admission. Cultures of the right shoulder eventually grew *Clostridium septicum*.**

***Conclusion:*** **It is imperative to consider clostridial gangrene in the differential diagnosis for any patient with cancer and a fever of unknown origin.** © 1994 Wiley-Liss, Inc.

KEY WORDS

*Clostridium* infections, gangrene, uterine neoplasms

Accounts of necrotizing infections of soft tissues are found in the writings of Hippocrates.[1] The first modern description is credited to Joseph Jones, a surgeon in the Confederate Army.[2] During the Civil War 2,642 soldiers developed gangrene in battlefield wounds with a mortality of 46%.

Necrotizing soft tissue infections may be classified as clostridial or nonclostridial.[3] Nonclostridial infections are polymicrobial and are believed to evolve through synergy between facultative and anaerobic bacteria.[4] Clostridial infections are usually monomicrobial, with *Clostridium welchii* (also known as *C. perfringens*) the most common isolate. *C. septicum* accounts for one third of all cases.[5]

Clostridial infections range from simple wound contaminations—organisms found in devitalized tissues without actual tissue invasion—to life-threatening myonecrosis (gangrene) characterized by invasion into and destruction of otherwise healthy tissues. Gangrene is often preceded by trauma such as crush injuries, penetrating wounds, compound fractures, or prolonged interruptions in arterial perfusion. Spontaneous, atraumatic cases of clostridial gangrene have recently been reported.[5,6]

The presence of *C. septicum* in necrotizing soft tissues infections, particularly the atraumatic type, may be increasing in incidence.[7] Of particular interest is the association between *C. septicum* gangrene and malignant disease.[8] We report herein the first known case of spontaneous, atraumatic *C. septicum* gangrene occurring in a patient with recurrent endometrial adenocarcinoma, emphasizing the

Address correspondence/reprint requests to Dr. David H. Moore, Department of Obstetrics and Gynecology, Indiana University Medical Center, 550 North University Boulevard, UH #2440, Indianapolis, IN 46202-5274.

Received January 12, 1994
Accepted March 23, 1994

clinical manifestations and management of clostridial myonecrosis.

### CASE REPORT

A 63-year-old white female with recurrent endometrial adenocarcinoma presented for evaluation of right shoulder pain. Three weeks prior to admission she became easily fatigued with normal daily activity. On the day prior to admission, the patient noted a dull, aching, nonradiating pain in her right shoulder described as an "arthritis-like" pain. The patient applied ice to her shoulder and took 50 mg of oxycodone without relief. On the morning of admission the pain was worse with the development of paresthesias and edema of the right shoulder. The patient's daughter noted a large black-blue discoloration on the right shoulder. Upon further questioning, she denied fever, chills, or trauma to the shoulder.

Her medical history was pertinent for uterine cancer. Six years ago, she presented with postmenopausal bleeding and dilatation and curettage revealed the presence of a poorly differentiated endometrial adenocarcinoma. She underwent a total abdominal hysterectomy and bilateral salpingo-oophorectomy with pelvic washings. Pathology demonstrated a poorly differentiated adenocarcinoma with foci of clear cell differentiation and penetration through one third of the myometrium. Pelvic cytology was positive for malignant cells. Pelvic radiation therapy was administered in 180 cGy daily fractions to a total dose of 5,040 cGy followed by daily oral medroxyprogesterone. She remained without evidence for cancer until 8 months before admission when on follow-up examination a 2-cm vaginal apex lesion proved to be recurrent disease. The patient received 5 courses of cisplatinum and doxorubicin. Disease progression was confirmed prior to the 6th treatment course when pelvic examination showed the vaginal mass to be increasing in size. The patient was started on tamoxifen and the 1st course of ifosfamide/mesna chemotherapy was given 1 month before admission.

Physical examination at time of admission revealed an ill-appearing, cachectic elderly woman supporting her right arm. She was afebrile and her blood pressure was 100/50. Her right shoulder and upper extremity were remarkable for an area of dark blue discoloration with crepitus (Fig. 1).

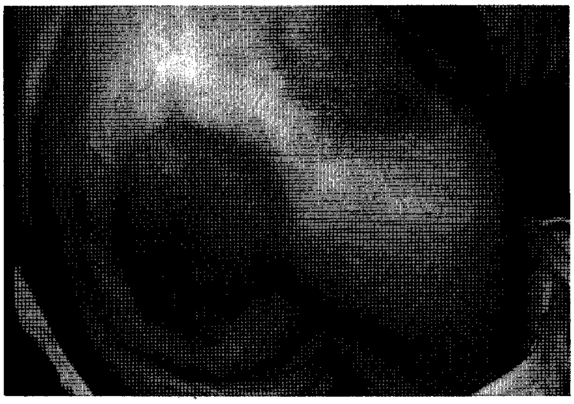

**Fig. 1. Two areas of discoloration are seen involving the right shoulder and represent cutaneous gangrene. Both lesions were crepitant to palpation.**

There was a drastic decrease in the range of motion of the right shoulder and the patient was unable to grip with her right hand. Pulses were diminished in the left arm and absent in the right upper extremity.

Laboratory studies at the time of admission included hemoglobin 8.3 g/dl; hematocrit 24.5%; WBC count 8,200/μl with 3% metamyelocytes, 58% bands, 31% polymorphonuclear leukocytes, and 8% monocytes. Other laboratory studies included sodium 127 meq/l; chloride 86 meq/l; blood urea nitrogen (BUN) 35 mg/dl; calcium 8.3 mg/dl; albumin 2.3 g/dl; SGOT 171 IU/l; alkaline phosphatase 171 IU/l; and CK 3,650 IU/l. Serum creatinine, platelet count, and prothrombin and partial thromboplastin times were normal. Chest and shoulder films were normal. Computed tomography of the thorax revealed gas in the right shoulder tissues.

Fluid was aspirated from the shoulder and submitted for Gram stain and culture. To distinguish between a primary vascular occlusion and nontraumatic myonecrosis, we ordered an angiogram which demonstrated diffuse narrowing of the arteries of the right upper extremity and diffuse soft tissue gas and edema consistent with gangrene. The Gram stain of the shoulder aspirate demonstrated gram-positive spore-forming rods with many polymorphonuclear leukocytes.

After contemplating her recurrent cancer and end-stage condition, the patient declined surgical intervention. She expired within hours of admission. Cultures of the right shoulder eventually grew *C. septicum*.

### DISCUSSION

Since the legalization of induced abortion, reports of clostridial myonecrosis/gangrene have become sparse in the gynecologic literature. Braverman and colleagues[9] reported a case of traumatic *C. septicum* uterine infection following curettage for endometrial cancer. A spontaneous case of *C. welchii* infection was reported by Lacey and colleagues[10] in a patient undergoing chemotherapy for choriocarcinoma. In both cases, cancer was the common denominator.

There were about a dozen cases of *C. septicum* infections reported to the National Communicable Disease Center between 1940 and 1967 vs. 27 cases between 1963 and 1968.[8] The prevalence of *C. septicum* in atraumatic cases of gangrene also seems to be increasing.[5,7,11] Reasons for the increasing incidence and prevalence of life-threatening *C. septicum* infections are unknown but may include better screening for the organism.[11] *C. septicum* is believed to be normal colonic flora; however, this is controversial due to the difficulty in isolating the organism from the stool of normal persons.[11] Clostridial species (including *C. septicum*) can be isolated from the vaginal secretions of 5–10% of asymptomatic women.[12] Therefore, *C. septicum* may be an opportunistic pathogen.

Associations between *C. septicum* infections and malignancy are increasingly recognized. Between 1977 and 1979, 7 patients were treated at Massachusetts General Hospital for either *C. septicum* bacteremia or gangrene and all had associated malignancies.[13] Alpern and Dowell[8] reported that 23/27 (85%) patients with *C. septicum* septicemia had malignant disease. The more common cancers associated with *C. septicum* infections include gastrointestinal, genitourinary, and hematologic malignancies. Tumors may provide direct portals for entry. *C. septicum* produces 4 exotoxins leading to necrosis of surrounding tissues. Upon penetrating normal tissue barriers, bacteria may enter the coelomic cavity or the bloodstream. In colon cancers, interruptions in colonic mucosal integrity provide an avenue for the intestinal organisms. Cancer chemotherapy may also be a predisposing factor for *C. septicum* infection. In one series, 14/23 (61%) patients with *C. septicum* infections complicating cancer were undergoing chemotherapy and 6 other patients had tumors involving the colon.[8] In leukemias, necrotizing enterocolitis during episodes of drug-induced neutropenia has been reported.[14] Chemotherapy may directly facilitate the entry of *C. septicum* through patchy necrosis of epithelial cells leading to interruptions in the colonic mucosa, or indirectly through damage resulting form mucosal hemorrhage during periods of drug-induced thrombocytopenia.[14] Prior to this report, only 4 other cases of clostridial sepsis in association with gynecologic cancers have been reported and all followed either surgical instrumentation or chemotherapy.[9,10]

The rapid clinical deterioration of our patient with spontaneous *C. septicum* myonecrosis is a hallmark of the disease. Mortality from clostridial septicemia ranges from 67 to 100%.[5,11,15] Bretzke and coworkers[16] showed that the mortality rate associated with *C. septicum* infections is 3 times higher than that associated with all other clostridial infections. The relative severity of *C. septicum* infections may be due to the greater aerotolerance of this organism.[5]

Successful treatment is dependent upon early diagnosis due to the rapid progression of the disease.[17] Pertinent symptoms and signs include the sudden onset of severe pain in a wound, often with a nonpurulent exudate, and localized edema. Diffuse bronzing of the skin, crepitus, cutaneous gangrene, or hemorrhagic bullae indicate advanced disease and therefore are poor prognostic signs. Tachycardia is a common systemic manifestation. Fever is often absent. The presence of hypotension portends impending cardiovascular collapse.

After diagnosis, the treatment plan includes 1) aggressive operative debridement; 2) adequate antibiotic therapy; and 3) supportive measures including fluid resuscitation and cardiovascular support. *C. septicum* is sensitive to large doses of penicillin G. Chloramphenicol or clindamycin may be used in the penicillin-allergic patient. Antibiotics are an adjunct, not a replacement for aggressive surgical debridement of devitalized tissues. Hyperbaric oxygen therapy prevents bacterial exotoxin production and may improve the clinical outcome. The effectiveness of polyvalent gangrene antitoxin is under investigation.

The link between malignant disease and *C. septicum* myonecrosis is recognized but poorly understood. Knowing that this association exists, one must consider clostridial gangrene in the differential diagnosis for any patient with cancer and a fever of unknown origin.

### REFERENCES

1. Finegold SM: Anaerobic Bacteria in Human Disease. New York: Academic Press, p 408, 1977.
2. Wilson B: Necrotizing fascitis. Am Surg 18:416–431, 1952.
3. Dellinger EP: Severe necrotizing soft-tissue infections: Multiple disease entities requiring a common approach. JAMA 246:1717–1721, 1981.
4. Giuliano A, Lewis F Jr., Hadley K, Blaisdell FW: Bacteriology of necrotizing fascitis. Am J Surg 134:52–57, 1977.
5. Stevens DL, Musher DM, Watson DA, Eddy H, Hamill RJ, Gyorkey F, Rosen H, Mader J: Spontaneous, nontraumatic gangrene due to *Clostridium septicum.* Rev Infect Dis 12:286–296, 1990.
6. Narula A, Khatib R: Characteristic manifestations of *Clostridium*-induced spontaneous gangrenous myositis. Scand J Infect Dis 17:291–294, 1985.
7. Jendrzejewski JW, Jones SR, Newcombe RL, Gilbert DN: Nontraumatic clostridial myonecrosis. Am J Med 65:542–546, 1978.
8. Alpern RJ, Dowell VR Jr: *Clostridium septicum* infections and malignancy. JAMA 209:385–388, 1969.
9. Braverman J, Adachi A, Lev-Gur M, Fallen S, Rosenzweig M, Greston WM, Kleiner GJ: Spontaneous clostridia gas gangrene of uterus associated with endometrial malignancy. Am J Obstet Gynecol 156:1205–1207, 1987.
10. Lacey CG, Futoran R, Morrow CP: *Clostridium perfringens* infection complicating chemotherapy for choriocarcinoma. Obstet Gynecol 47:337–341, 1976.
11. Kornbluth AA, Danzig JB, Bernstein LH: *Clostridium septicum* infection and associated malignancy. Report of 2 cases and review of the literature. Medicine 68:30–37, 1989.
12. Sweet RL, Gibbs RS: Infectious Diseases of the Female Genital Tract. Baltimore: Williams & Wilkins, p 5, 1985.
13. Case Records of the Massachusetts General Hospital: Case 49-1979. N Engl J Med 301:1276–1281, 1979.
14. King A, Rampling A, Wight DGD, Warren RE: Neutropenic enterocolitis due to *Clostridium septicum* infection. J Clin Pathol 37:335–343, 1984.
15. Case Records of the Massachusetts General Hospital: Case 46-1990. N Engl J Med 323:1406—1412, 1990.
16. Bretzke ML, Bubrick MP, Hitchcock CR: Diffuse spreading *Clostridium septicum* infection, malignant disease and immune suppression. Surg Gynecol Obstet 166: 197–199, 1988.
17. Corey EC: Nontraumatic gas gangrene: Case report and review of emergency therapeutics. J Emerg Med 9:431–436, 1991.
